# Visualizing profitability: A heatmap approach to evaluate Bitcoin futures trading using VMA trading rules

**DOI:** 10.1016/j.heliyon.2023.e21376

**Published:** 2023-10-21

**Authors:** Min-Yuh Day, Yensen Ni, Chinning Hsu, Paoyu Huang

**Affiliations:** a Graduate Institute of Information Management, National Taipei University, Taipei, Taiwan; b Department of Management Sciences, Tamkang University, New Taipei, Taiwan; c Department of International Business, Soochow University, Taipei, Taiwan

**Keywords:** Bitcoin futures, VMA trading rules, Heatmap visualization, Technical analysis, Trading performance

## Abstract

Given that technical trading charts are publicly available on popular financial websites such as Bloomberg and MarketWatch, it stands to reason that the same technical trading approaches may be applied to cryptocurrency markets. One of these trading strategies is the variable length moving average (VMA), whose flexibility benefit has not been fully explored in prior research. To fill this gap, we evaluate Bitcoin futures using VMA trading rules and provide the results in a heatmap diagram. This approach allows investors to choose the most effective VMA rules, potentially leading to profits. Furthermore, our approach may shed new light on previously unexplored investment thinking and practices that have the potential to improve investment outcomes.

## 1 Introduction

According to the efficient market hypothesis, stock prices may incorporate all available information [1,2], making it difficult to forecast stock prices. Nonetheless, this perspective appears to be challenged by phenomena like disposition effects [3], the overreaction hypothesis [4], and even herding behaviors [5,6]. While trading in stock markets, many investors employ technical trading rules, such as contrarian technical trading regulations (e.g., relative strength index) based on the overreaction hypothesis [7], momentum technical trading regulations (e.g., moving average) based on stock price trends [8], and even excessive self-confidence [9]. Among a variety of technical trading rules, moving average (MA) trading rules are widely used in stock markets [[10], [11], [12]]; however, variable length moving average (VMA) trading rules with the flexibility merit are infrequently fully considered in the relevant studies.

In this study, we use Bitcoin futures instead of stocks as our investigated objective because Bitcoin has received increasing media and investor attention due to its innovation, transparency, simplicity, and rising popularity [13]. Bitcoin has garnered the most attention among cryptocurrencies [14,15] due to the challenging process of transferring large sums of money [14,15]. However, its value is extremely volatile, making it unreliable as a form of payment in the financial payment system.

Furthermore, in the captivating realm of cryptocurrency trading research, a constellation of studies unveils the secrets to success [16]. uncovered the potency of moving average strategies, with Dash shining brightly amidst privacy coins. Transitioning to Bitcoin [17], harnessed the power of deep learning, particularly recurrent neural networks, to decode the market's complexities [18]. dissected Bitcoin's returns, revealing the prowess of technical analysis on a daily horizon, with the formidable random forest as the ultimate predictor. Additionally, regarding the valuation of Bitcoin [19], introduced a groundbreaking non-parametric model, with neural networks as the ultimate forecasting tool [20]. unlocked the secrets of Bitcoin trading, with the trading range breakout strategy outshining traditional methods. Finally [21], utilized deep learning to enhance Bitcoin intraday trading, demonstrating resilience even during challenging market conditions. These studies illuminate a path to success in the dynamic cryptocurrency landscape, where innovation and data-driven insights reign supreme.

Moreover, from the perspective of macroeconomics, Bitcoin can be viewed as a hedge against rising inflation [22,23], a financial instrument incorporating a portfolio [24], and even a safe haven against economies. Due to the characteristics of high risk and high expected return, lower transaction fees, stealth, and the convenience of cross-border trading, Bitcoin became a popular “currency,” attracting a wide variety of investors and even individual investors [25,26].

To familiarize ourselves with pertinent studies, we find that the effectiveness of MA trading rules has been confirmed by relevant studies [10,[27], [28], [29]], which may contradict the efficient market hypothesis. Due to the ongoing debate over the effectiveness of technical analysis, the issues pertaining to the effectiveness of technical trading rules may require additional investigation. As a result of surveying the relevant studies in terms of MA and VMA trading rules and heatmap visualization in the literature review section, this study addresses the research gap by examining the underexplored area of variable length moving average (VMA) trading rules in Bitcoin futures trading, offering flexibility not extensively studied in the context of stock markets where moving average (MA) trading rules are more common. This study then seeks to shed light on the effectiveness of VMA rules and their potential impact on trading outcomes, providing valuable insights into a relatively unexplored area of financial research. Additionally, we argue that this study investigates the following novel aspects that have been understudied in the relevant literature. First, we extend the applicability of MA trading strategies by investigating the flexibility of VMA rules in Bitcoin futures trading, thus expanding the spectrum of potential trading outcomes. Second, we enable investors to recognize superior performance areas within a heatmap matrix, thereby enhancing their capacity for decision-making. Third, our innovative heatmap-based approach to Bitcoin futures trading is a promising strategy for achieving superior results. Therefore, by leveraging VMA trading insights, diversifying outcomes, and augmenting decision-making through visualization, our study enriches the literature and increases profitability in this domain.

As such, we contend that this study may add to the existing body of knowledge in a variety of ways. First, due to the flexibility merit of the VMA trading rules, we can derive numerous outcomes by utilizing numerous VMA trading regulations, which appear to be seldom considered in the relevant studies; additionally, this study will not only broaden the application scenarios for MA trading strategies, but it will also provide a variety of outcomes for market participants interested in trading Bitcoin futures. Second, by trading Bitcoin futures according to various VMA trading regulations, we demonstrate that investors are able to identify the better performance area among a variety of outcomes shown in a heatmap matrix, which is likely to benefit these investors who can select better outcomes among numerous outcomes. Third, we believe that many investors would be interested in our novel research methodology since, as compared to the prior design, using a heatmap diagram for trading Bitcoin futures might produce much superior results. This study may not only improve the profitability of trading Bitcoin futures but also benefit investment practices that were previously unaddressed by using the wisdom of VMA regulations, obtaining a number of outcomes utilizing a variety of VMA, observing overall outcomes via heatmap visualization, and even determining how to derive much better outcomes among these various outcomes.

## 2 Literature review

This study investigates whether investors who implement multiple VMA trading rules would profit from trading Bitcoin futures utilizing heatmap visualization. Since we introduced and reviewed the pertinent literature on cryptocurrencies in the previous section, in this section we introduce MA and VMA trading rules, as well as heatmap visualization, and review their relevant studies.

### 2.1 MA trading rules

Concerning MA trading rules, we find that their effectiveness has been supported by relevant studies [10,[27], [28], [29], [30], [31]]. Nonetheless, given the ongoing debate over the effectiveness of technical analysis, it is possible that the issue of the effectiveness of technical trading rules is still unresolved and thus deserves further investigation.

Relevant research investigates the MA trading strategy based on the interaction of short-term MA (SMA) and long-term MA (LMA) lines, which is commonly used in practice. For example, the MA trading rule is used to trade stocks in U.S. markets [9,32] and global financial markets [28,33,34].

Over the past decade, a body of research has emerged, offering insights into the efficacy of the MA trading rule [35]. uncovered its ability to predict future stock values through trading signals like golden or dead crosses [36]. attributed the popularity of the MA trading rule to its capacity to identify price momentum and exploit price autocorrelation structures efficiently. [37] extended these findings, demonstrating the rule's superior forecasting power in emerging equity markets. More recently [38], showcased the dominance of neural networks incorporating the MA trading rule, surpassing other machine learning techniques in predictive accuracy. However [39], cautioned that positive returns for market participants employing technical trading rules may diminish when transaction costs are considered. Additionally [40], recommended the utilization of the MA trading rule for investors in less developed markets marked by informational inefficiencies, highlighting its potential advantages in such environments.

### 2.2 VMA and FMA trading rules

The vast majority of research on technical analysis focuses on MA trading rules [10,27,28,[33], [34]]. As per [10]; the most widespread MA strategy is 1–200, which generates buying (selling) signs as the 1-day MA advances (declines) the 200-day MA. In addition, previous research suggests that investors who use diverse MA trading rules (i.e., variable length moving average (VMA) and fixed length moving average (FMA)) would be profitable [10,11,[27], [41]].

According to FMA trading rules [11], investors are advised to purchase and hold their trading objectives (e.g., stocks, index futures, etc.) for a fixed time to measure their performance. However, we argue that there may not be a criterion for selecting a certain time frame as the exit for such rules, as opposed to VMA trading rules with the obvious entry (exit) (i.e., golden crosses (dead crosses)) [42]. demonstrated that VMA trading regulations possess some predictive capability [43]. found that VMA trading rules outperform other technical trading rules when evaluating and forecasting the efficacy of technical trading rules in cryptocurrency markets. Consequently, we investigate whether investors can profit from trading Bitcoin futures using a variety of VMA trading regulations.

In contrast to 1–100 and 1–200 [10,27], many practitioners use 5–20, 5–60, and 20–60 [10,27]. Therefore, we employ the 5–20, 5–60, and 20–60 MA trading regulations, as they appear to be utilized frequently by market participants. We believe that by combining different VMA (SMA (day x), LMA (day y)) (hereafter abbreviated as VMA (x, y)) with y > x, this design can produce additional profit opportunities. As shown in Table 3 (i.e., a number of results generated using diverse VMA regulations) or Fig. 2 (i.e., a number of results visualized using a heatmap), the objective of this study is to identify the outcome with the highest profitability when trading Bitcoin futures using numerous VMA trading rules.

Furthermore, we indicate that some investors employ one of the MA trading regulations, such as (1,50), (1100), and (1,150), where the first number in parentheses is the SMA and the second number is the LMA. Then, investors may discover that one of the MA trading rules for trading financial instruments has the best performance when compared to the other MA trading rules, which may be considered the traditional design for evaluating various MA trading rules [10,27]. However, due to the flexibility of VMA trading rules, we can obtain numerous outcomes based on various days utilized by either SMA or LMA, and these numerous outcomes will be displayed in heatmap visualization. Thus, in contrast to the traditional designs described previously, we not only present all of the results within a matrix of heatmaps, but we also disclose which VMA trading rule would produce the best results, or which special area would produce superior outcomes in the heatmap matrix.

### 2.3 Heatmap data visualization technique

A heatmap is a frequently used data visualization technique in artificial intelligence [44,45] and big data analytics [46,47]. For instance, [48] argue that heatmap visualization techniques can aid in elucidating the visual data processing predictions of deep artificial intelligence. Using the heatmap technique [49], constructs a pricing model to identify the significant variables on Airbnb sharing economy rental platforms. Despite extensive computer science literature on heatmap data visualization, few empirical finance studies use it to present and analyze financial findings. Consequently, we contend that investors can evaluate all of the outcomes by this technique, which may assist them in selecting the best outcome and even selecting a specific area whose results are superior to the best outcome shown in conventional design.

## 3 Design of this study

### 3.1 MA and VMA trading rules

According to MA trading regulations, market participants are advised to buy (sell) stocks when a golden (dead) cross occurs (i.e., when the SMA crosses the LMA upwards (downwards), a buy (sell) signal is generated). Technical analysts and practitioners can employ VMA trading regulations based on the various days utilized by the SMA or LMA. Consequently, numerous outcomes (i.e., a number of VMA (x, y) with y > x) would be obtained, primarily due to the flexibility advantage of using days for SMA and LMA.

### 3.2 Research design

Initially, this study utilizes several VMA trading regulations including VMA (5, 20), VMA (5, 60), and VMA (20, 60) since 5-, 20-, and 60-day MAs are employed in practice as weekly, monthly, and quarterly MAs. As part of this study's conventional design, we also include semiannual and prolonged MA (e.g., VMA (5, 120), VMA (5, 180), VMA (20, 120), and VMA (20, 180). In addition to the traditional design, our new design allows us to derive a variety of outcomes by using different days for SMA and LMA, as shown in Table 3 (i.e., VMA (x, y), where x is from 5 to 60, y is from 10 to 180, and y > x) or Fig. 2 (i.e., all of the outcomes shown in a heatmap diagram).

Furthermore, to avoid limited transactions that could skew our results, we chose 60 and 180 days for x and y with 5-day intervals. Moreover, despite our best efforts to present our findings, the aforementioned concern may be the limitation of this study due to the absence of a standardized presentation of our findings.

### 3.3 Measuring rate of return based on VMA trading rules

The Bitcoin futures index return **(hereafter referred to as BR)** is then calculated based on the VMA trading regulations for Bitcoin futures using Equation (1).

(1) $$BR_{i}=\left(\alpha_{i}∕\beta_{i}\right)\;–\;1$$

where $\alpha_{i}$ = the closing Bitcoin futures index on the day of exit at the i-th trade

$\beta_{i}$ = the closing Bitcoin futures index on the day of entry at the i-th trade

The aggregate Bitcoin futures returns **(hereafter referred to as CBR)** are calculated using Equation (2).

(2) $$CBR=\sum_{i=1}^{n}(1+BR_{1})\left(1+BR_{2}\right)..........\left(1+BR_{n}\right)$$

Following that, using Equation (3), the geometric mean of the Bitcoin futures index return (**hereafter referred to as GMBR**) can be calculated.

(3) $$GMBR=\sqrt[{n}]{CBR}$$

After executing the initial round-trip trade, traders move on to the subsequent round-trip trade. Traders then apply this trading method sequentially from the initial trade (i) to the final trade (n). Since we can calculate the next BR after determining the previous BR, we contend that using the geometric mean is more suitable for this study than employing the arithmetic mean.

Despite this, investors who trade Bitcoin futures should consider transaction fees^1^; round-trip trading Bitcoin futures costs 0.04 % or less (e.g., 0.017 %). The maximum is more than 28 %, and the minimum is more than 1 %, according to the GMBR in Table 2 (i.e., the outcomes of the traditional approach). Consequently, the transaction fee could be insignificant.

**Table 1:** Descriptive statistics.

Crypto Currencies	Sample	Mean	SD.	CV	Median	MIN	MAX
Bitcoin futures	1824	6135.05	4762.74	77.63 %	6410.25	364.33	29374.15

Table 1 displays the mean, standard deviation (SD), coefficient of variation (CV), median, maximum (MIN), and minimum (MAX) of the Bitcoin futures index from 2016 to 2020.

**Table 2:** Results for the traditional design.

(1)	(2)	(3)	(4)	(5)	(6)	(7)
VMA rules	Total number of trades	CBR (%)	GMBR (%)	CV	Mean DA	Maximum DA
(5, 20)	95	4196.63	4.05	6.82	19	74
(5, 60)	40	2830.87	8.83	15.13	41	156
(5, 120)	18	8687.96	**28.26**	**35.16**	89	478
(5, 180)	10	196.12	12.12	15.23	106	249
(20, 60)	28	607.20	7.35	9.15	58	192
(20, 120)	10	253.28	13.48	17.48	160	477
(20, 180)	6	17.92	1.38	8.18	170	386

Table 2 presents the mean, VMA strategies, the total number of trades, CBR%, GMBR%, coefficient of variance (CV), mean duration day (DA), and maximum (DA) for the data period 2016–2020.

**Table 3:** GMBR results derived from adopting numerous VMA trading rules in a heatmap matrix.

180	12.1	−9.3	−13.0	1.4	−10.0	2.1	10.9	4.4	7.5	3.5	15.9	13.8
175	6.9	−11.7	−10.6	−7.6	−10.4	3.7	14.1	1.0	10.2	4.8	13.1	10.4
170	6.6	−7.8	−9.5	−6.0	−13.2	−5.0	16.1	5.8	11.6	7.0	14.8	10.0
165	5.5	−7.5	−8.2	−6.8	−12.9	0.5	14.2	6.9	13.3	10.8	14.5	14.6
160	7.4	−8.4	−3.4	−6.3	−9.9	2.9	14.2	5.7	13.7	12.0	15.2	14.3
155	12.5	−8.8	−5.1	−8.4	−6.8	5.6	13.8	4.5	13.2	11.9	52.1*	49.7*
150	10.7	−10.5	−6.1	−9.8	−7.6	6.6	10.7	44.4*	50.9*	45.8*	48.3*	50.1*
145	12.8	−6.6	−4.8	3.2	33.2*	44.4*	56.1***	33.7*	54.1*	49.3*	52.7*	47.8*
140	10.4	−5.0	23.6*	18.9*	38.5*	44.6*	56.1**	54.6*	58.0*	58.3*	51.7*	47.6*
135	27.1*	18.0*	24.0*	17.9*	22.8*	36.6*	56.3**	56.7*	59.9*	61.3*	60.4*	52.7*
130	27.3*	21.9*	16.0*	11.0	22.1*	38.5*	56.8**	61.1*	60.5*	60.8*	61.6*	61.1*
125	32.6*	21.7*	23.6*	14.8*	41.7*	30.4*	54.3*	63.2*	66.2*	59.6*	61.2*	59.1*
120	28.3*	21.7*	34.8*	13.5*	41.5*	36.8*	55.9*	62.3*	65.8*	63.6*	58.5*	48.9*
115	26.7*	16.9*	29.7*	12.4*	32.6*	36.5*	45.9*	63.5*	68.4*	44.8*	47.5*	46.8*
110	22.6*	21.8*	28.9*	14.6*	31.4*	33.5*	46.5**	62.8*	54.0*	44.5*	49.1*	44.5*
105	22.6*	17.4*	18.6*	14.9*	17.9*	32.3*	48.3*	44.8*	51.4*	47.8*	50.4*	41.4*
100	27.5*	16.2*	19.1*	15.9*	28.3*	32.8*	30.0*	35.5*	38.3*	32.4*	35.2*	33.1*
95	23.6*	11.0*	15.4*	25.8*	22.9*	25.4*	28.0*	27.8*	30.1*	28.9*	30.2*	29.0*
90	13.3*	22.7*	14.9*	18.1*	7.2*	7.0*	19.3*	17.5*	25.2*	29.4*	24.1*	25.0*
85	15.4*	19.4*	19.5*	12.2*	7.6*	1.4	13.5*	17.0*	15.8*	23.1*	23.7*	15.9
80	14.3*	14.7*	18.1*	9.3*	14.2*	13.7*	7.6*	13.9*	17.4*	12.9	17.9*	18.1*
75	10.0*	15.6*	12.7*	9.1*	13.2*	12.6*	11.0*	5.8*	13.6*	8.7*	11.9*	9.7*
70	10.1 *	13.3	12.5	18.0*	5.6	9.0	12.3*	9.4*	0.8	3.5	5.9 *	5.3
65	10.6 **	10.6*	10.8*	10.6*	11.3 *	6.7	3.9	2.7	2.0	1.6	3.8	1.7
60	8.8 *	10.2*	10.7*	7.4	6.8	1.5	2.9	1.2	−0.6	3.1	1.7	
55	11.0 **	8.0*	7.9*	7.6*	6.3*	2.7	3.7	3.0	−0.6	1.3		
50	8.6*	9.5*	7.9*	8.8	4.2	8.0*	4.0	3.0	2.7			
45	5.7*	10.5 **	12.7 **	8.6*	5.1	7.0*	5.4*	0.6				
40	6.6*	9.9*	8.6 *	8.0*	8.4*	6.1*	4.6*					
35	6.9*	10.7 **	9.6*	6.3*	5.8*	3.2						
30	5.3*	8.3*	9.5*	7.4*	3.8							
25	5.8 ***	8.5	5.4*	3.4*								
20	4.0*	5.6*	4.9*									
15	2.0*	1.5*										
10	0.2											
n2/n1	5	10	15	20	25	30	35	40	45	50	55	60

Note: The cells are the of VMA (x, y) trading rules, where x in red is from 5 days to 60 days in the last row of Table 3, y in blue is from 10 days to 180 days in the first column of Table 2, and n2>n1, and each cell in the heatmap matrix presents the GMBR derived using one of diverse VMA (x, y) regulations. The red cells in the heatmap matrix have over 40 % GMBR. Additionally, *P＜0.05.

Moreover, it is possible to establish an acceptable criterion for evaluating the results of Bitcoin futures trading. The annual yield on one-year Treasury bills usually reflects the potential loss (i.e., opportunity cost) of using cash as an investment. Moreover, the performance of the S&P 500 index can be used as a benchmark because it may represent the performance of trading index ETFs that track the performance of the S&P 500 index. Thus, the performance of the S&P 500 index could be compared to that of trading Bitcoin futures utilizing numerous VMA trading rules.

## 4 Empirical results and analyses

### 4.1 Descriptive statistics

**Fig. 1:**
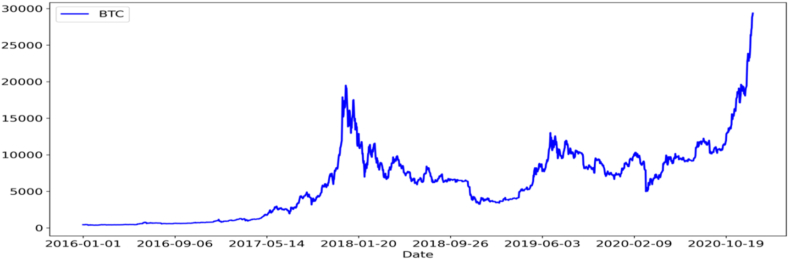
The trend of the Bitcoin futures index from 2016 to 2020.

Our samples consist of daily Bitcoin futures index information from Datastream. Table 1 displays the mean, median, standard deviation (SD), coefficient of variance (CV), minimum (MIN), and maximum (MAX) values for the Bitcoin futures index from 2016 to 2020. Table 1 shows that the difference between the MAX and MIN is quite large, indicating that the Bitcoin futures index's movement is quite volatile, as evidenced by the high SD shown in Table 1. In addition, after plotting the Bitcoin futures index data, Fig. 1 displays a peak near the beginning of 2018 and a sharp rise near the end of the data period.

**Fig. 2:**
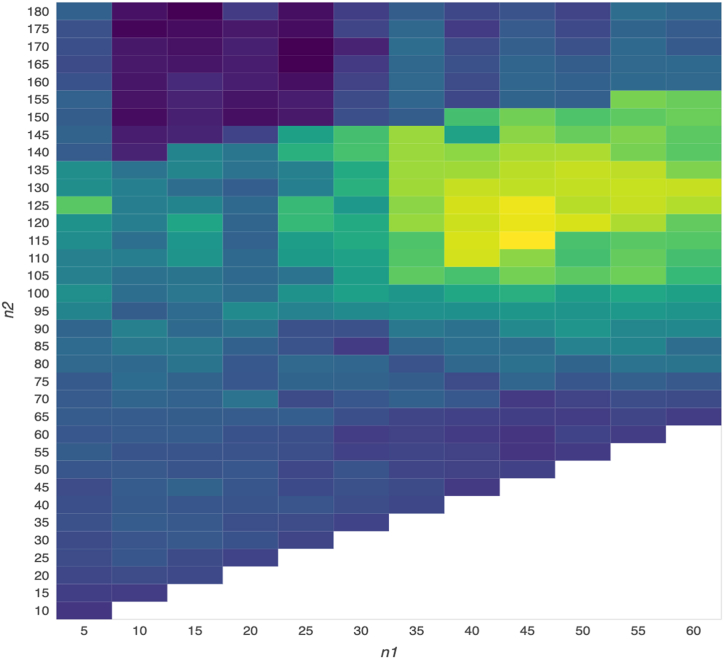
GMBR outcomes derived adopting a number of VMA regulations.

### 4.2 Results of the traditional design

In addition to the previously mentioned VMA (5, 20), VMA (5, 60), and VMA (20, 60), our traditional design also employs seminal MA and even extended MA, such as VMA (5, 120), VMA (5, 180), VMA (20, 120), and VMA (20, 120). Table 2 displays the results.

Table 2 shows that by utilizing the VMA (5, 120) trading regulation, investors can obtain a GMBR of 28.26 %, that has been far greater than GMBRs obtained by utilizing other VMA trading regulations (i.e., all other GMBRs in Table 2), indicating that the highest return is also accompanied by the highest risk (i.e., the highest CV shown in Table 2). For reference, Table 2 also includes the total number of trades, average duration day, and maximum duration day for each VMA trading rule. However, we argue that investors would prefer much higher returns than the maximum GMBR using the traditional approach. Consequently, we employ our new design, as shown in the following section.

### 4.3 Results representing a heatmap diagram

Thus, investors can utilize a range of VMA (SMA (day x), LMA (day y) regulations to yield potentially much higher GMBRs, as shown in a heatmap matrix in Table 3. Then, we present the numerous results (GMBRs) in this matrix that incorporate a number of VMA results; additionally, each cell in the heatmap matrix represents the GMBR obtained by applying one of these VMA (x, y) trading rules.

Following that, after examining the performance of various VMA trading rules in Table 3, we find that the GMBRs in red are all greater than 40 %, which is significantly higher than the highest return of the traditional design (i.e., 28.26 %); these higher GMBRs are also displayed in the cells with a bright yellow color in Fig. 2.

As a result, by implementing our new design, investors may be able to choose much more suitable VMA regulations to generate higher profits, as shown in the red area (i.e., higher than 40 %), even substantially higher profits in this red area (e.g., there are a number of combinations whose s are greater than 60 % within this red area). Our remarkable results can be attributed to the use of big data analytics and the adaptability of VMA trading rules, both of which are incorporated into our new design.

Furthermore, when we compare the outcomes presented in Table 3, Table 4, we observe some consistent findings. Specifically, the regions highlighted in red in Table 3 exhibit notably high Sharpe ratios in Table 4. This suggests that our findings are not only reflected in terms of returns (as demonstrated by GMBRs in Table 3) but also in risk-adjusted returns (as indicated by the Sharpe ratio results in Table 4). This consistency emphasizes the durability of our results when measured using a different performance criterion.

**Table 4:** Sharpe ratio results derived from adopting numerous VMA trading rules in a heatmap matrix.

180	0.08	−0.09	−0.07	0.01	−0.09	0.02	0.08	0.04	0.08	0.04	0.08	0.09
175	0.07	−0.09	−0.08	−0.07	−0.09	0.03	0.08	0.01	0.07	0.05	0.07	0.08
170	0.09	−0.09	−0.10	−0.07	−0.08	−0.05	0.07	0.06	0.06	0.07	0.08	0.06
165	0.08	−0.08	−0.09	−0.08	−0.07	0.01	0.08	0.07	0.07	0.06	0.07	0.08
160	0.08	−0.10	−0.04	−0.07	−0.09	0.03	0.05	0.06	0.09	0.08	0.08	0.09
155	0.06	−0.09	−0.06	−0.09	−0.07	0.05	0.04	0.05	0.15	0.12	0.11	0.11
150	0.05	−0.06	−0.07	−0.08	−0.08	0.06	0.12	0.10	0.11	0.08	0.10	0.13
145	0.08	−0.08	−0.06	0.04	0.08	0.09	0.10	0.08	0.11	0.10	0.10	0.11
140	0.07	−0.06	0.07	0.05	0.09	0.09	0.11	0.12	0.13	0.13	0.10	0.11
135	0.08	0.06	0.07	0.05	0.05	0.09	0.12	0.11	0.13	0.13	0.12	0.11
130	0.08	0.06	0.05	0.03	0.05	0.09	0.12	0.12	0.13	0.13	0.12	0.11
125	0.09	0.06	0.08	0.04	0.10	0.08	0.13	0.11	0.12	0.13	0.12	0.11
120	0.09	0.06	0.09	0.04	0.11	0.09	0.13	0.12	0.12	0.13	0.13	0.11
115	0.09	0.05	0.08	0.04	0.09	0.09	0.13	0.14	0.13	0.09	0.11	0.11
110	0.08	0.05	0.08	0.04	0.10	0.10	0.12	0.14	0.11	0.09	0.10	0.11
105	0.07	0.05	0.05	0.04	0.05	0.09	0.12	0.11	0.12	0.10	0.10	0.10
100	0.06	0.07	0.05	0.04	0.07	0.08	0.09	0.11	0.10	0.08	0.08	0.08
95	0.05	0.06	0.07	0.06	0.06	0.09	0.09	0.08	0.10	0.09	0.08	0.07
90	0.08	0.05	0.07	0.05	0.02	0.02	0.07	0.06	0.08	0.09	0.07	0.07
85	0.07	0.08	0.08	0.03	0.02	0.00	0.04	0.06	0.06	0.08	0.08	0.05
80	0.06	0.09	0.09	0.05	0.08	0.08	0.05	0.09	0.07	0.05	0.07	0.06
75	0.05	0.09	0.08	0.05	0.07	0.07	0.06	0.04	0.08	0.09	0.07	0.08
70	0.04	0.05	0.06	0.08	0.07	0.07	0.07	0.08	0.01	0.07	0.06	0.07
65	0.08	0.08	0.09	0.09	0.08	0.08	0.08	0.06	0.04	0.04	0.09	0.04
60	0.05	0.08	0.09	0.04	0.09	0.03	0.06	0.03	−0.01	0.07	0.05	
55	0.08	0.06	0.06	0.06	0.07	0.05	0.08	0.06	−0.01	0.03		
50	0.08	0.05	0.06	0.08	0.09	0.07	0.08	0.06	0.07			
45	0.08	0.07	0.08	0.08	0.09	0.05	0.07	0.01				
40	0.07	0.06	0.06	0.06	0.08	0.05	0.07					
35	0.05	0.08	0.08	0.07	0.07	0.08						
30	0.07	0.07	0.07	0.05	0.08							
25	0.06	0.06	0.08	0.09								
20	0.07	0.08	0.06									
15	0.07	0.08										
10	0.01											
n2/n1	5	10	15	20	25	30	35	40	45	50	55	60

Note: The cells are the of VMA (x, y) trading rules, where x in red is from 5 days to 60 days in the last row of Table 4, y in blue is from 10 days to 180 days in the first column of Table 4, and n2>n1, and each cell in the heatmap matrix presents the Sharp ratio derived using one of these diverse VMA (x, y) regulations.

## 5 Concluding remarks

### 5.1 Conclusions and discussion

Numerous well-known financial websites (e.g., MarketWatch, Investopedia, TradingView, and Bloomberg) display trading stocks according to MA trading rules from a practical perspective. In the relevant research literature, the profitability of employing the MA trading rule is also extensively examined from an academic perspective. Consequently, this study indicates that the efficiency of MA trading rules merits further study, as many investors are interested in whether it is possible to profit by trading financial instruments using the MA trading rule.

In addition, due to the flexibility of using various days for SMA and LMA, this study focuses primarily on the VMA trading rule. In addition to the traditional design, our new design investigates whether investors can obtain significantly higher returns, including the highest return, by either evaluating a number of results obtained using diverse VMA regulations or by observing all of the outcomes displayed in a heatmap diagram.

As such, our study makes several key findings that may contribute to the existing literature as follows. First, we investigate the application of various VMA (Variable Moving Average) trading rules in the context of trading Bitcoin futures and their representation in heatmap diagrams. This aspect of VMA trading rules has received limited attention in prior research, which often focuses on technical indicators like annualized returns and abnormal returns [9,30].

Second, our findings suggest that investors trading Bitcoin futures using VMA rules can identify superior performance areas within heatmap diagrams. This empowers investors to select VMA trading rules based on the specific performance areas that matter most, in contrast to previous studies that typically present overall results without highlighting performance in specific areas [41].

Third, we propose that our research design, employing heatmap diagrams for Bitcoin futures trading, could offer enhanced performance compared to traditional approaches. While heatmap visualization techniques are commonly used in fields like computer science [44,45] and big data analytics to analyze two-dimensional data matrices, their application in finance, especially for financial data and big data, remains limited. Therefore, our study not only advances result presentation in finance but also provides a valuable tool for investors to make more informed decisions by visualizing the overall outcome through heatmap diagrams.

To summarize, this study employs numerous VMA investing rules based on big data analytics, employs a heatmap visualization technique to evaluate the profitability of employing numerous VMA rules, and then applies such technique in investment practice, all of which may contribute to the existing literature.

### 5.2 Research implications

First, we contend that this study has significant implications for investors. Using VMA technical trading regulations to assess the trading performance of Bitcoin futures, we first present a novel strategy for cryptocurrency trading. Using a heatmap to illustrate our results, this cutting-edge technique yields novel market insights. Our method offers cryptocurrency investors a practical method for selecting VMA trading rules with the potential to generate profits. The use of heatmaps to analyze results is a user-friendly technique that facilitates the selection process. Compared to conventional designs, we believe that our heatmap diagram has the potential to generate significantly improved results in trading Bitcoin futures, thereby benefiting both investment practices and existing literature.

Second, this study proposes that by using a variety of VMA trading regulations, investors can achieve higher returns. By presenting results in a clear heatmap visualization, investors can easily identify the most profitable outcomes. Moreover, investors can make decisions prior to implementing numerous trading regulations by utilizing big data analytics This study aims to provide valuable insights for enhancing Bitcoin futures trading profitability, as preparation and well-informed decision-making are key success factors, particularly when trading Bitcoin futures with a high level of leverage risk.

Third, we provide an innovative solution for cryptocurrency market investors. By providing this innovative and user-friendly method, we may aid investors in making well-informed decisions, minimizing investment risk, and achieving market-beating returns. Moreover, our novel approach sheds light on the significance of technical trading rules in the cryptocurrency industry and emphasizes an understudied aspect of the flexibility of VMA trading rules. This study lays the groundwork for future research on the implementation of VMA technical trading rules in cryptocurrency markets and can be used as a guide for investment practices in this rapidly evolving field.

### 5.3 Limitation and future research

There are numerous limitations to the examination of VMA regulations that should be noted. To begin, while the study sets various VMA regulations, there is still an opportunity for improvement by increasing the variable lag lengths (n1 ranging from 5 to over 60 and n2 ranging from 10 to over 180) or adopting a shorter period, such as 2.5 rather than 5. This may result in additional information and outcomes. Second, due to employing Bitcoin futures instead of Bitcoin spots, we then assume that traders with abundant capital might not have to concern margin amount resulting from the volatilities of futures prices in this study, which would be another limitation of this study. Third, because the data period exhibited an upward trend, the study's findings were obtained from bull markets. As a result, it is unknown if these strategies would be profitable during bear markets. It would be interesting to compare the results of the Bitcoin futures index's bull and bear market periods in future studies. Furthermore, future research could look into other potential avenues. In addition to Bitcoin futures, the study could look at the performance of other cryptocurrencies as investment targets. Additionally, the research design might be expanded to include financial instruments such as stocks and ETFs, as well as commodity assets such as gold and oil, to evaluate if the novel design outperforms the previous approach in trading these assets.

## Declaration of competing interest

The authors declare that they have no known competing financial interests or personal relationships that could have appeared to influence the work reported in this paper.

## Footnotes

1 https://bitflyer.com/en-jp/commission.
